# Molecular fluorescence spectroscopy with multi-way analysis techniques detects spectral variations distinguishing uninfected serum versus dengue or chikungunya viral infected samples

**DOI:** 10.1038/s41598-020-70811-7

**Published:** 2020-08-13

**Authors:** Marfran C. D. Santos, Joelma D. Monteiro, Josélio M. G. Araújo, Kássio M. G. Lima

**Affiliations:** 1 Biological Chemistry and Chemometrics, Institute of Chemistry, Federal University of Rio Grande do Norte, Natal, 59072-970 Brazil; 2 Laboratory of Molecular Biology for Infectious Diseases and Cancer, Department of Microbiology and Parasitology, Federal University of Rio Grande do Norte, 59072-970 Natal, Brazil; 3 Laboratory of Virology, Institute of Tropical Medicine, Federal University of Rio Grande do Norte, 59072-970 Natal, Brazil

**Keywords:** Microbiology, Analytical chemistry, Cheminformatics

## Abstract

Significant attempts are being made worldwide in an attempt to develop a tool that, with a simple analysis, is capable of distinguishing between different arboviruses. Herein, we employ molecular fluorescence spectroscopy as a sensitive and specific rapid tool, with simple methodology response, capable of identifying spectral variations between serum samples with or without the dengue or chikungunya viruses. Towards this, excitation emission matrices (EEM) of clinical samples from patients with dengue or chikungunya, in addition to uninfected controls, were separated into a training or test set and analysed using multi-way classification models such as n-PLSDA, PARAFAC-LDA and PARAFAC-QDA. Results were evaluated based on calculations of accuracy, sensitivity, specificity and F score; the most efficient model was identified to be PARAFAC-QDA, whereby 100% was obtained for all figures of merit. QDA was able to predict all samples in the test set based on the scores present in the factors selected by PARAFAC. The loadings obtained by PARAFAC can be used in future studies to prove the direct or indirect relationship of spectral changes caused by the presence of these viruses. This study demonstrates that molecular fluorescence spectroscopy has a greater capacity to detect spectral variations related to the presence of such viruses when compared to more conventional techniques.

## Introduction

The dengue virus (DENV) and the chikungunya virus (CHIKV) are considered the most important arboviruses in the world based on the number of human infections that occur annually; they are transmitted by mosquitoes of the genus *Aedes*^1^. The DENV belongs to the *Flaviviridae* family, *Flavivirus* genus, the same as other well-known viruses such as Zika and Yellow Fever. Currently, there are four known serotypes of Dengue (DENV-1, DENV-2, DENV-3, DENV-4). An individual previously infected by one of these serotypes is immune to this serotype for the rest of their life, but they remain susceptible to other serotypes of Dengue^2^. The CHIKV belong to the *Alphavirus* genus*, Togaviridae family*. The symptoms caused due to infections by these arboviruses are clinically similar, with acute onset fever, joint pain, rash, fatigue, muscle pain and headache^3^. Therefore, when sensitive and specific detection techniques are not used, their diagnosis becomes inaccurate. Chikungunya and dengue infections are spatio-temporally related and may even occur as co-infections^4^.

The primary diagnostic methods for arboviruses can be classified into two main groups: direct or indirect^5^. Direct methods are more sensitive, specific, complex and expensive; hence, they are only employed in leading hospitals and clinics or in virological study centres. Amongst the direct methods, there are those that use viral isolation, genome detection (PCR) and antigen detection. On the other hand, indirect methods are less sensitive and specific; however, they provide faster results, are easier to process and less expensive, so they are more readily found in diagnostic clinics. Amongst the indirect methods, there are the so-called "rapid tests" and the serological tests that detect IgM and IgG antibodies. Indirect methods with immunoenzymatic assays can allow the occurrence of cross-reactions when other arboviruses are co-circulating. This is because the surface proteins of such viruses are similar, causing the immune system to produce nonspecific sub-neutralizing antibodies and causing serological techniques to generate inaccurate diagnosis^6,7^. Due to the wide spectrum of symptoms that arboviruses can cause, it is necessary that in addition to clinical diagnosis, there is also a laboratory diagnosis in order to differentiate one infection from another. Some such methods performed in laboratories are: viral isolation, molecular techniques, serological tests and rapid tests^8^.

Viral isolation (gold standard method), as well as reverse transcription techniques followed by polymerase chain reaction (RT-PCR) and real-time RT-PCR (qRT-PCR) are commonly performed in the viremia phase to detect viral genetic material because they are very sensitive^9–12^, especially in serum samples, and the most specific diagnostic approaches^13^. Imunoenzymatic tests such as ELISA (Enzyme-Linked Immunosorbent Assays) to detect antibodies of the IgM and IgG immunoglobulin classes are widely used in the diagnosis of infections^14,15^. Currently, there are several rapid tests available on the market towards detection of antigens and/or antibodies allowing quick and low-cost diagnosis. However, rapid tests should only be used as an initial screening tool for arbovirus infections. Limitations such as the high occurrence of cross-reactions (low specificity) require an additional assessment by molecular techniques such as RT-PCR or qRT-PCR^16^.

When it comes to the standard techniques used for the diagnosis of these arboviruses, in the document *"Tool for the diagnosis and care of patients with suspected arboviral diseases*" published by the World Health Organization (WHO) in 2017, the limitations are clear. For patients with arbovirus symptoms where, for example, DENV, zika virus and CHIKV circulate, three RT-PCR analyses are necessary to make a conclusive diagnosis. However, this approach is expensive and requires primers (specific kits to diagnose each type of virus). Therefore, it is critical to develop a simpler, cheaper and faster diagnostic technique^17^.

Studies in the literature involving biospectroscopy with chemometrics applied in virology are rare, especially involving molecular fluorescence spectroscopy. We identified no studies in the field of virology where EMM spectroscopy was used together with classification algorithms, demonstrating the novelty of our approach. Studies involving mid-infrared (MIR), near-infrared (NIR) or Raman spectroscopy are available. Recently, ATR-FTIR spectroscopy was used with PCA-LDA, SPA-LDA or GA-LDA to classify samples from patients diagnosed with dengue, zika or chikungunya virus versus uninfected controls. The results demonstrate the great potential of the technique for this type of classification, where results up to 100% specificity and sensitivity are observed^18^. A recent study shows the potential of ATR-FTIR spectroscopy with the same classification techniques in discriminating serum samples infected with different viral loads of DENV-3, with up to 100% accuracy obtained^19^. In both studies, the time between obtaining samples and acquiring spectra was relatively the same (1 year between obtaining the first sample and acquiring the spectra).

Molecular fluorescence spectroscopy is known as one of the most sensitive spectroscopic techniques, being capable of detecting chemical species at very low concentrations. This is an important advantage in the field of viral detection since the viral load in some patients may be minimal. It is also a relatively inexpensive technique compared to other spectroscopic techniques. Fluorescence excitation–emission matrices (EEM) contain a large amount of information on fluorophores (analytes capable of emitting fluorescence signals). The EEM matrix is obtained by acquiring a number x of emission spectra for n excitations. Through these matrices, chemometric analyses can be performed to classify samples using second-order analysis techniques^20^.

EEM matrices can be unfolded (U) to vectors, where well-known 1st-order classification algorithms such as principal component analysis (uPCA), successive projection algorithm (uSPA), genetic algorithm (uGA) and partial least squares (uPLS) can be applied together with linear/quadratic discriminant analysis (LDA or QDA). Alternatively, three-way data cubes can be used with 2nd-order techniques such as parallel factor analysis (PARAFAC) in conjunction with LDA or QDA and nway-partial least squares-discriminant analysis (n-PLSDA) for classification. Herein, 2nd-order data (EEM matrices) were used. In this approach, PARAFAC can be employed to extract more redundant information (scores and loadings) from the data while reducing its size, whereas LDA or QDA use this extracted information to find linear (LDA) or quadratic (QDA) functions that better discriminate the classes. n-PLSDA is an extension for 2nd-order data from the well-known PLSDA widely used for 1st-order data. In n-PLSDA, latent variables (LVs) with highly-observed covariance of the data are selected. The scores present in these LVs can be used in the classification process. These algorithms are amongst the most applied chemometric techniques to analyse biological data, where their potential together with EEM matrices was previously studied in the discrimination between *Cryptococcus neoformans* and *Cryptococcus gattii* pathogenic fungi^21^.

In the present study, EEM matrices of serum samples from 26 uninfected individuals, 26 patients with DENV and 26 patients with CHIKV were analysed by multi-way classification algorithms (PARAFAC-LDA, PARAFAC-QDA or n-PLSDA), in order to evaluate the potential of molecular fluorescence spectroscopy for classification of these serum samples. This is a pioneering study on the use of EEM fluorescence spectroscopy with chemometric techniques for the detection of arboviruses.

## Results

**Figure 1:**
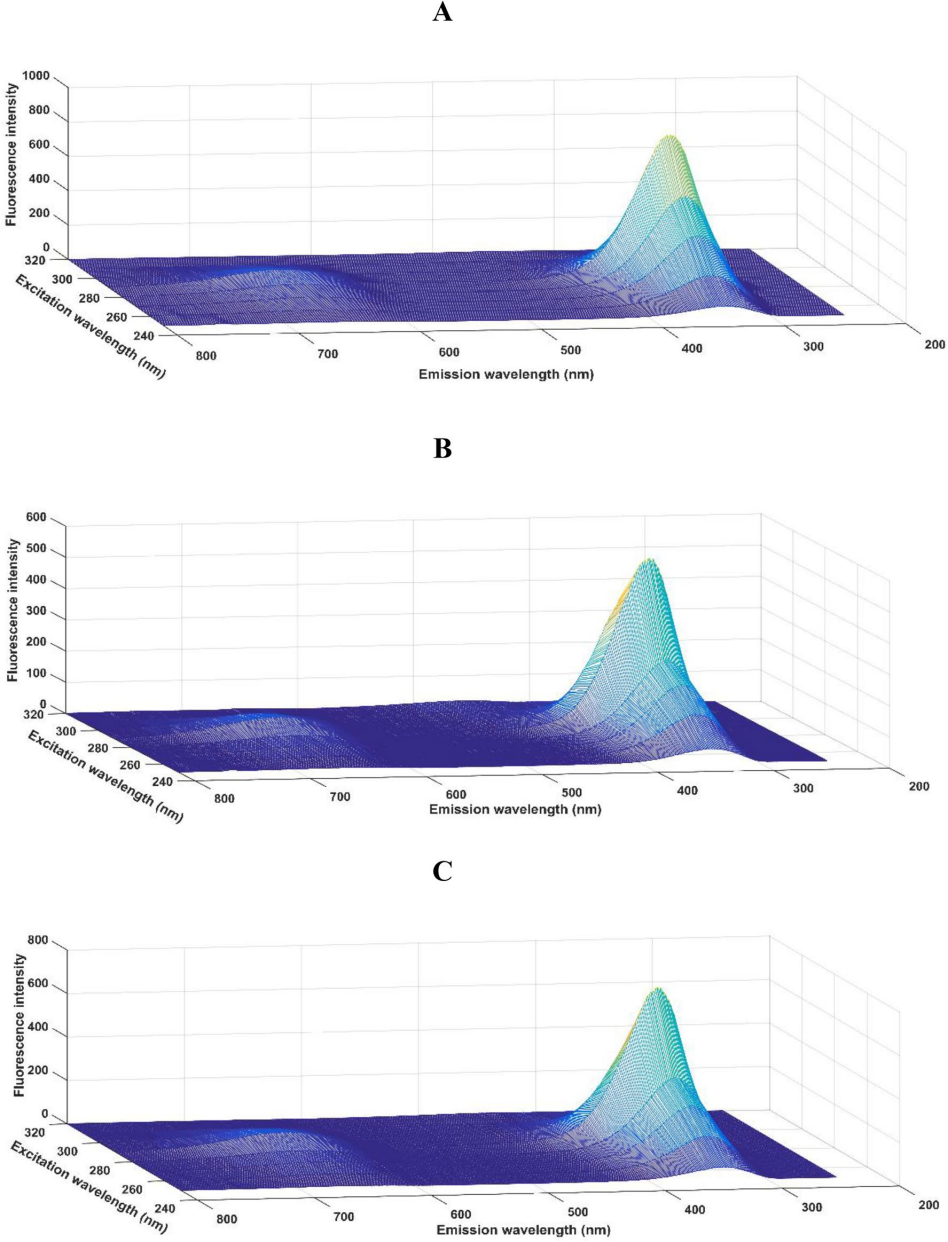
Excitation–emission molecular fluorescence spectra obtained for clinical serum samples: (**a**) uninfected; (**b**) with DENV; and, (**c**) with CHIKV. Rayleigh and Raman were removed from the spectra. The excitation/emission wavelength range was 250–320 nm for excitation and 240–800 nm for emission, with steps of 10 and 1 nm, respectively.

Figure 1 shows the excitation–emission matrix (EEM) fluorescence spectrum of one sample of each class after removing Rayleigh and Raman scatterings. Figure 1a shows the EEM spectrum for an uninfected sample, Fig. 1b for a DENV sample and 1c for a CHIKV sample. As depicted in Fig. 1a–c, there is marked similarity between the spectra indicating a high spectral overlapping of the data when plotted together, making it difficult to distinguish them. For this reason, it is necessary to use techniques capable of maximizing the differences between the samples of different classes.

Seventy-eight (n = 78) samples were used in this study, 26 for each class, which were divided into training (54 samples, 18 from each class) and test (24 samples, 8 from each class) sets. Table 1 shows the results in terms of correct classification rate (CC%) of the classification models using n-PLSDA, PARAFAC-LDA or PARAFAC-QDA with the EEM fluorescence matrices towards discriminating between uninfected, DENV and CHIKV serum samples.

**Table 1:** Correct classification rates obtained for n-PLSDA, PARAFAC-LDA and PARAFAC-QDA classification models between Uninfected, DENV and CHIKV.

Model	Class	CC training (%)	CC test (%)
n-PLSDA (6)^a^	Uninfected	78.57	66.66
	DENV	71.42	83.33
	CHIKV	92.85	100.0
PARAFAC-LDA (3)^b^	Uninfected	85.71	66.66
	DENV	92.85	100.0
	CHIKV	100.0	100.0
PARAFAC-QDA (3)^b^	Uninfected	100.0	100.0
	DENV	100.0	100.0
	CHIKV	100.0	100.0

The CC% represents the percentage of samples correctly classified, considering their true classes. The calculation is made based on Eq. (8) (see “Quality performance”), where ε_1_ represents class 1 errors (class of interest) and ε_2_ represents class 2 errors (all samples from another class).

^a^Number of latent variables; ^b^number of parallel factors.

### n-PLSDA

**Figure 2:**
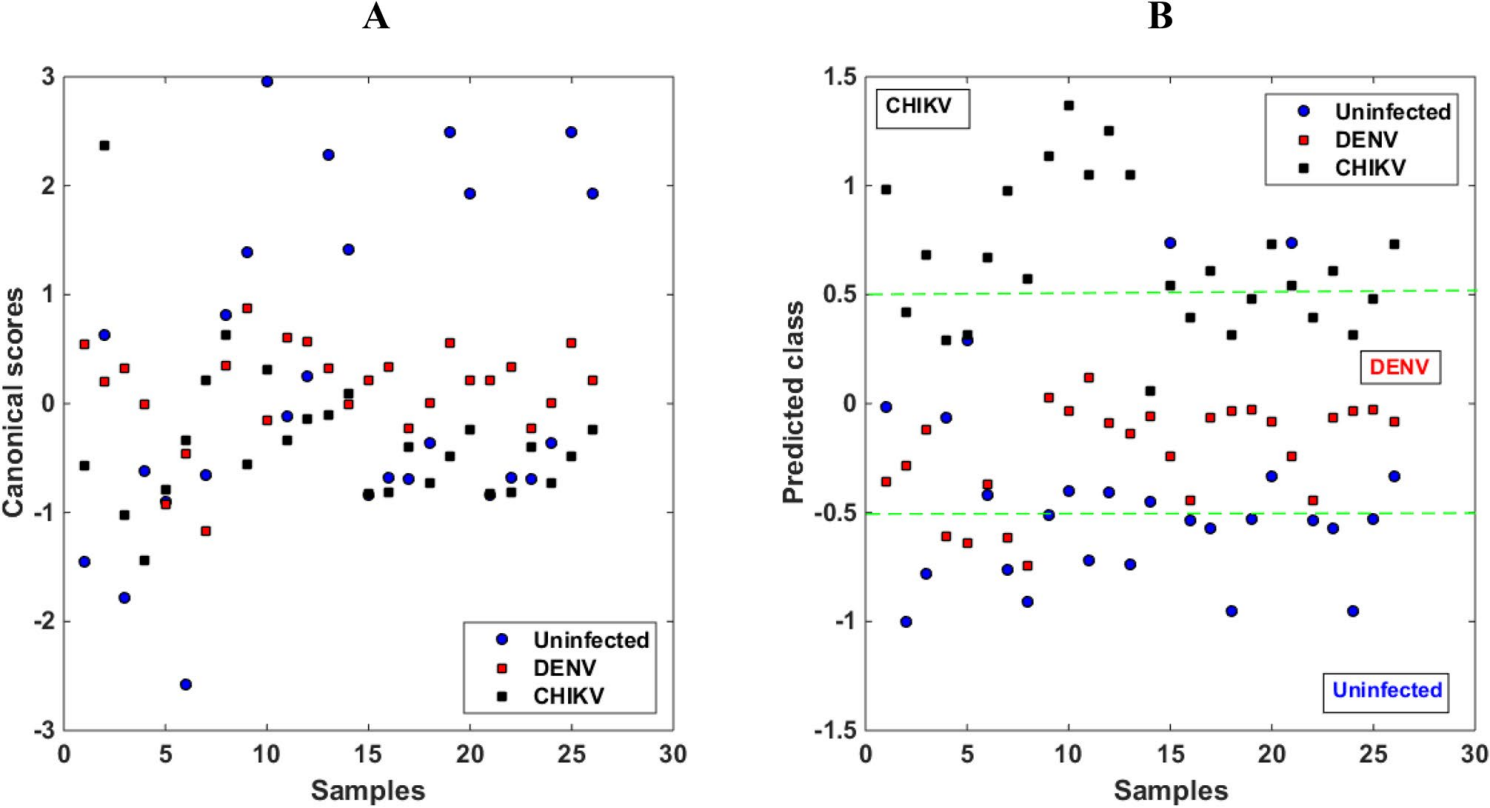
(**a**) Canonical scores of the n-PLSDA for the 2 main latent variables; and, (**b**) predicted class values. The points refer to clinical samples of uninfected serum (blue circle), with DENV (red square), and with CHIKV (black square).

The n-PLSDA model used a data cube size 78 × 8 × 561. The model was constructed with 6 latent variables based on SVD, accounting for 97% of the data-defined variance. As can be seen in Table 1, in the training stage the correct classification rate was higher for serum samples from patients with CHIKV, the second best rate of correct classification was obtained for serum samples from uninfected individuals, and the lowest correct classification rate in training was observed for serum samples from patients with DENV. In the test stage, the correct classification rate was lower for uninfected samples, and it increased for DENV or CHIKV samples. Figure 2 shows the canonical scores of the n-PLSDA on the two first latent variables (Fig. 2a) and the predicted class values (Fig. 2b).

Subsequently, discriminant models based on LDA and QDA were applied in an attempt to maximize the differences between samples of different classes and thus improve the classification results.

### PARAFAC-LDA and PARAFAC-QDA

**Figure 3:**
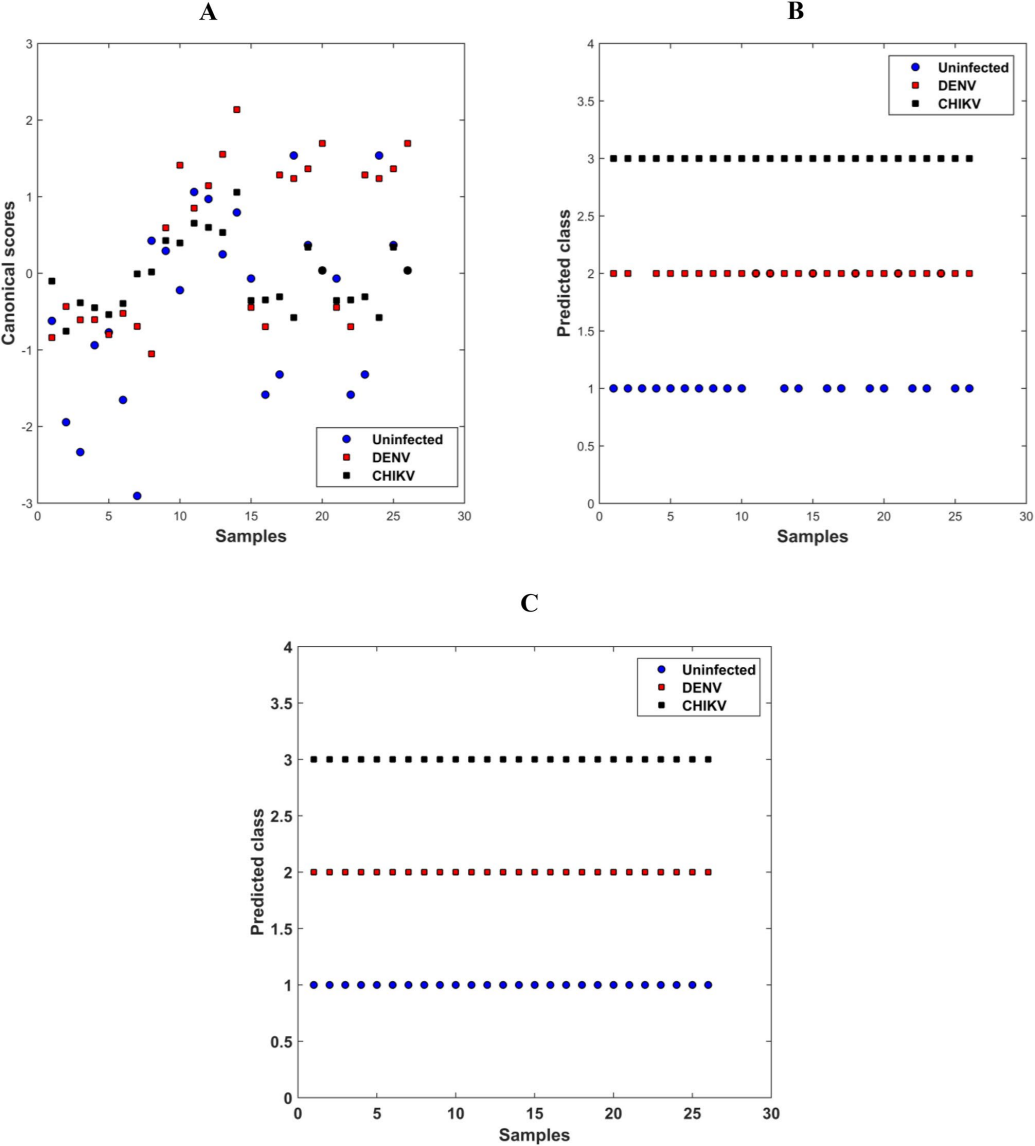
(**a**) Canonical scores of the PARAFAC; (**b**) predicted class values by PARAFAC-LDA; and, (**c**) predicted class values by PARAFAC-QDA. The points refer to clinical samples of uninfected serum (blue circle), with DENV (red square), and with CHIKV (black square).

PARAFAC was built using only 3 factors, which explained 98% of the observed data variance. The scores obtained in these 3 factors were used as input data in the LDA and QDA classification. The correct classification rates for PARAFAC-LDA and PARAFAC-QDA are shown in Table 1. Figure 3 shows the canonical scores of the two first factors of the PARAFAC model (Fig. 3a), the predicted class values by PARAFAC-LDA (Fig. 3b), and the predicted class values by PARAFAC-QDA.

To validate the study, figures of merit (accuracy, sensitivity, specificity and F score) were calculated. Table 2 shows the validation values of the optimized n-PLSDA, PARAFAC-LDA and PARAFAC-QDA models for each class. The calculations were made based on Eqs. 9, (10), (11) and (12) (“Quality performance” below), which are based on the values of TP, TN, FP and FN obtained in the test stage.

**Table 2:** Figures of merit for the models n-PLSDA, PARAFAC-LDA and PARAFAC-QDA applied to emission-excitation matrices of serum samples.

Figures of merit	Models		
	n-PLSDA	PARAFAC-LDA	PARAFAC-QDA
**Uninfected**			
Accuracy	78.57	88.88	100.0
Sensitivity	87.50	100.0	100.0
Specificity	66.66	66.66	100.0
F score	75.67	80.0	100.0
**DENV**			
Accuracy	81.25	88.88	100.0
Sensitivity	80.0	83.33	100.0
Specificity	83.33	100.00	100.0
F score	81.63	90.90	100.0
**CHIKV**			
Accuracy	83.33	100.0	100.0
Sensitivity	75.0	100.0	100.0
Specificity	100.0	100.0	100.0
F score	85.71	100.0	100.0

The calculations of the figures of merit are based on Eqs. (9), (10), (11) and (12) (see “Quality performance”), and considers only the test step.

**Figure 4:**
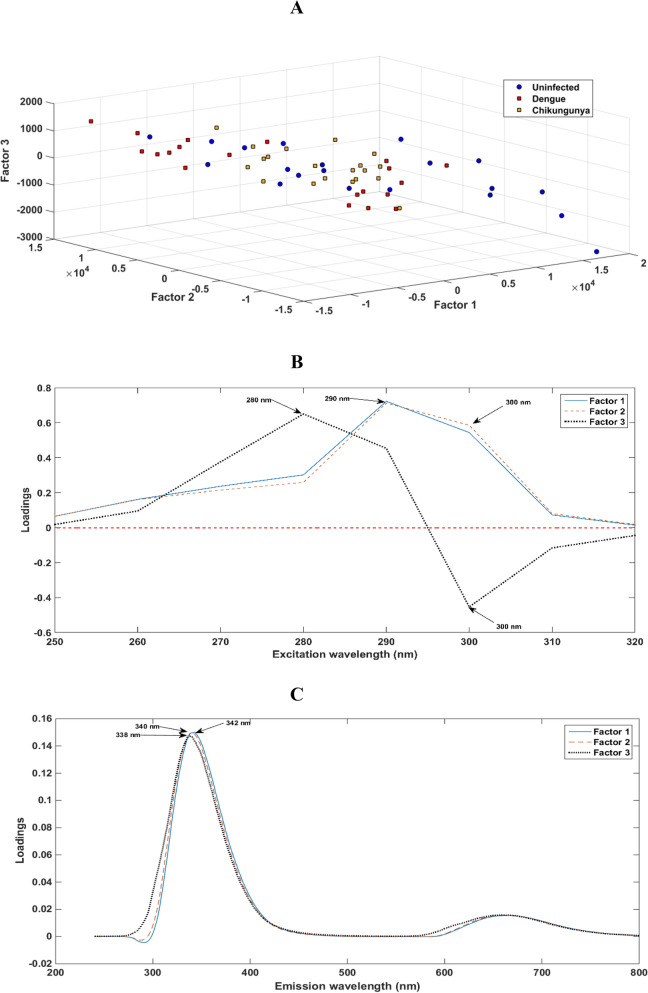
Scores and loadings on FAC 1, FAC 2 and FAC 3 selected in PARAFAC. (**a**) Scores; (**b**) loadings for excitation; and, (**c**) loadings for emission. In the PARAFAC model, a decomposition of the data is condensed into trilinear factors (or FAC). Each FAC consists of a scoring vector and two loading vectors.

Figure 4 shows the scores (Fig. 4a), loadings for excitation (Fig. 4b) and loadings for emission (Fig. 4c) on the 3 factors used for the construction of the PARAFAC-LDA and PARAFAC-QDA models.

## Discussion

When analysing the EMM spectra of uninfected serum samples (Fig. 1a) and samples with viruses (Fig. 1b,c), it is possible to observe a considerable decrease in the intensity of the fluorescence signal in the range between 300 and 415 nm. This may occur as a consequence of chemical virus–cell interactions. In these spectra, the highest peak is characteristic of endogenous fluorophores, such as ADP, ATP, GTP, AMP, NADH, glutathione oxide, oxidized glutathione, reduced glutathione, acetyl-CoA, structural proteins (collagen and elastin), amino acids (tryptophan, tyrosine, phenylalanine) and others^22^. In addition, the lowest peak may be related to a wide variety of organic compounds such as vitamins and lipids, which may exhibit auto-fluorescence in this region^23^.

Spectral changes related to pathophysiological changes that can affect the classification result are reduced in the sampling process. This is because in the Kennard–Stone (KS) algorithm, the samples with greater dissimilarities (more distant from the centre of their class) are kept in the training set (about 70% of the samples), while the others are kept in test. Even if it is not possible to know what is responsible for the similarities and differences found by the KS algorithm, we know that the samples that most differ are found in the training set. Consequently, once there are pathophysiological changes, the samples of major changes will be in the training set. Therefore, the test set will contain samples with minor pathophysiological changes and, of course, with the respective changes in each class (uninfected samples, samples with DENV and samples with CHIKV). This reduces the contributions of pathophysiological changes in the classification work.

The results of CC% for n-PLSDA shown in Table 1 can be considered good since in the test stage, values ranging from 83.33 to 100.0% are observed for DENV and CHIKV, respectively; however, the values observed for uninfected serum samples, especially at the testing stage, are unsatisfactory. This suggests that the model is not well fitted (the model was unable to extract scores that had a correlation with the samples’ features capable of differentiating them), and perhaps a larger number of uninfected samples would be needed for model construction. In fact, it has already been mentioned in the literature that PLS-based models require a larger number of samples for the residual matrix to accommodate the least amount of information possible^21^. Therefore, even using EEM matrices that are 2nd-order data that contain more information than 1st-order data, it is observed that the n-PLSDA model did not obtain the ideal fit. Figure 2 shows the canonical scores of the n-PLSDA on the two main latent variables (Fig. 2a) and the predicted class values (Fig. 2b). The figure shows that the samples from the three classes are mixed, and analysing Fig. 2b (predicted classes), many uninfected samples are located in the sample space of CHIKV and, mainly, of DENV, which confirms the low classification performance of the model in the test set for uninfected samples. Therefore, the n-PLSDA model is not efficient for discriminating uninfected controls, DENV and CHIKV samples based on the number of samples provided. Probably, a larger sample number would enhance the training model, resulting in a better fit and making the model more efficient towards test samples.

In Table 1, it is observed that the results obtained by PARAFAC-LDA are better compared to those obtained by n-PLSDA. PARAFAC-LDA shows a greater efficiency in the classification both in the training and in the test sets, mainly for the CHIKV class that obtained CC% of 100% in both subsets. Finally, PARAFAC-QDA obtained CC% of 100% for all classes in both training and test stages, demonstrating an excellent model fit. Figure 3 shows how the samples are mixed only based on the factors (before classification), demonstrating that exploratory analysis alone is not enough to discriminate the samples. However, after the application of LDA or QDA, a higher correct classification rate is observed for PARAFAC-LDA and, especially, for PARAFAC-QDA, which correctly classifies all samples provided (Fig. 3c).

The accuracy values inform the number of correct predictions, considering true and false negatives. The observed values in Table 2 are encouraging, especially in relation to PARAFAC-QDA, which obtained 100% accuracy in relation to uninfected samples. The accuracy for the DENV class for all models was > 80%, with the best accuracy obtained by the PARAFAC-QDA model and the lowest accuracy value obtained by n-PLSDA (81.25% accuracy). The same trend is observed for the CHIKV class, with the lowest accuracy being obtained by n-PLSDA (83.33%), and both PARAFAC-LDA and PARAFAC-QDA obtaining maximum accuracy. The sensitivity obtained for the uninfected class also showed that the models based on PARAFAC demonstrate a greater predictive capacity than the n-PLSDA model. The values obtained for CHIKV were 75.0%, 100.0% and 100.0%. These values inform the percentage of positive samples correctly classified by the models. The specificity informs the percentage of negative samples that were correctly classified by the models. Table 2 also shows how the PARAFAC-LDA model managed to maintain a high specificity, with 100% for the three classes. The same is observed for the F score. Thus, the models based on PARAFAC have, in general, best figures of merit figures for classification based on the presence or absence of DENV or CHIKV in serum, when compared with n-PLSDA. This reinforces the fact that the n-PLSDA model needs more samples to be better adjusted, while PARAFAC-QDA achieves a better fit of the model with fewer samples^21^. On the other hand, comparing the PARAFAC-LDA and PARAFAC-QDA models, PARAFAC-QDA is the most successful model for classification. This is because LDA assumes a single covariance matrix for both classes, unlike QDA, which uses a variance–covariance matrix for each class for discrimination. Therefore, we can predict that, given the complexity of the data, the variance–covariance matrix of samples from different classes has considerable differences, thus being poorly explained by the LDA approach^24^.

The scores shown in Fig. 4a can be interpreted as the relative concentration of the analyte in the sample. These scores serve as codes that, when plotted, allow an exploratory analysis of the data. Note that only exploratory analysis of scores is not able to segregate classes. The loadings depicted in Fig. 4b,c represent the most important wavelengths for the factors used in the construction of the PARAFAC-LDA and PARAFAC-QDA models applied herein. Thus, the peaks present in the loadings graphs can be understood as “biological markers”. Loadings are also interpreted as the individual profiles of species that are emitting fluorescence signals. By analysing the loadings, typical peaks of endogenous fluorophores are observed. In the excitation loadings, the most relevant excitations are 280 nm, 290 nm and 300 nm. In this region, endogenous fluorophores tend to have a higher emission. In the emission loadings, the highest peak is in the emission range of fluorophores such as ADP, ATP, GTP, AMP, NADH, glutathione oxide, oxidized glutathione, reduced glutathione, acetyl-CoA, structural proteins (collagen and elastin), amino acids (tryptophan, tyrosine, phenylalanine) and others. The smallest peak is related to organic compounds, such as vitamins and lipids, which can exhibit auto-fluorescence in this range^22,23^.

The best relationship between the observed variation in the data and the number of factors (FAC) was found for three factors. That is, the addition of the fourth factor did not have a considerable increase in explained variance. This is in line with the optimization performed, where it is observed that when choosing 3 FACs, the lowest cross-validation error rate is obtained. Therefore, the first 3 FACs were selected and, consequently, three excitation and emission loadings were provided by PARAFAC. Since, in theory, the loadings are the individual spectral profiles of the species that are contributing to the excitation and emission signals, each group studied may have contributed a factor. That is, each load profile must be related to each class (uninfected, DENV and CHIKV). We suggest that the observed changes are related to viruses because the main difference between patients was the presence of viruses (DENV or CHIKV) or the absence of these viruses for uninfected individuals. However, this relationship can be direct or indirect. In addition to this, we suggest that the differences cannot be related to pathophysiological changes since some models obtained 100% accuracy in the test stage. The probability that individuals of a certain class had exactly the same pathophysiological similarities to each other is minimal. The fact that the model obtained a high correct classification rate suggests that these considerations are correct. Therefore, the changes must be associated with the presence of different chemical virus–cell interactions. In addition, the results show that discrimination is possible even if UV radiation causes damage to viral structures (which should not occur in an aqueous medium).

A disadvantage of using molecular fluorescence in this type of approach is the scarcity of studies involving the use of structure emission spectra of the DENV and CHIKV that makes it impossible to identify which species or interactions contribute to the loadings used in PARAFAC-QDA. Unlike the approach using ATR-FTIR spectroscopy, where several studies have already been done, and the region between 1,800 and 900 cm^−1^ is already known as the fingerprint region of biological samples; thus, it is possible to associate the most important wavenumbers shown by the loadings profiles with the biochemical structures of the virus^18,19,25^. However, the loadings herein suggest that the most important excitations for the construction of the factors, consequently for discrimination between classes, were those at 280 nm, 290 nm and 300 nm; and, the most important emission wavelengths were 338 nm, 340 nm and 342 nm. Therefore, further studies must be carried out to confirm the relationship between these wavelengths to some structure associated with the viral particles, which are mainly composed of a lipid bilayer, proteins and genetic material or relate them to alterations caused by virus–cell interaction.

The high sensitivity of molecular fluorescence spectroscopy has been described in the literature. Whether in books, as in Skoog's Fundamentals of Analytical Chemistry^26^, in which it states that "one of the most attractive characteristics of molecular fluorescence is its intrinsic sensitivity … among the types of compounds that can be determined by fluorescence are amino acids, proteins, coenzymes, vitamins, nucleic acids…". In fact, single molecules have already been detected by fluorescence spectroscopy, as observed by Peck et al.^27^. Therefore, the fact that single molecules have already been detected and, proteins and nucleic acids can be determined by molecular fluorescence demonstrates that viruses can also be determined, since they are composed mainly of proteins and RNA. It is also a fact that the fluorescence technique may not have the ideal specificity, however, it has a high sensitivity. This means that it is possible to readily detect variations in the sample composition. Additionally, one must analyse the specificity of the technique together with chemometric tools. Therefore, even if the specificity of the instrument is not ideal, when coupled with PARAFAC-QDA, for example, a specificity of 100% was observed. In a way, any minimal variation observed in the spectra must be increased when using n-PLSDA, PARAFAC-LDA or PARAFAC-QDA. This is a reason for using classification techniques. The role of LDA and QDA, as described, is to increase the differences between samples of different classes and to decrease the differences between samples of the same class. This improves sensitivity and specificity, as demonstrated herein.

Our results are encouraging considering that the figures of merit, mainly for the PARAFAC-QDA model, are compared to the gold standard technique used in the field of viral diagnosis. The calculations of the figures of merit used to validate the study demonstrate the applicability of this chemometric method for classification based on the presence or absence of these arboviruses. The results of sensitivity and specificity obtained by PARAFAC-QDA are equal to those of the gold standard method (viral isolation) currently used. Methods based on viral isolation have 100% sensitivity^1,2,5^. However, the methodology applied here has the advantage of obtaining the response for two viruses in just one analysis. When compared with indirect methods (most used in diagnostic clinics and hospitals), the results of sensitivity and specificities obtained by the worst model (n-PLSDA) are already comparable. Methods based on IgM detection can have sensitivity ranging from 21 to 99% and specificity ranging from 77 to 98%^28^. Consequently, PARAFAC-QDA gave higher sensitivity and specificity results than conventional diagnostic techniques (indirect methods). Another advantage of using molecular fluorescence is the fact that it does not require the use of reagents, unlike conventional techniques. Response time is also an advantage of the molecular fluorescence technique. The EEM matrix can be obtained in about 45 s, while a gold standard technique, for example, can take more than a week^28^. Additionally, when compared with the results obtained by ATR-FTIR spectroscopy with multivariate analysis techniques, both techniques achieved 100% sensitivity and specificity for uninfected clinical samples, DENV and CHIKV^18^. However, the molecular fluorescence approach has the advantage of even cheaper instrumentation when compared to ATR-FTIR spectroscopy.

Finally, this study demonstrates the potential of EEM spectroscopy together with multi-way analysis to differentiate clinical samples from uninfected serum, serum with DENV or with CHIKV, opening the future possibility of using these tools as a faster, more accurate and cheaper methodology. The most important excitation and emission wavelengths for class discrimination are provided in the loadings profiles. New studies must be carried out in order to associate this information with biochemical structures present in viruses, or with existing variations as a result of virus–cell interactions. Therefore, loadings are an important starting point. However, we can conclude that this study pioneers the use of molecular fluorescence spectroscopy for the rapid and simultaneous detection of DENV and CHIKV, presenting an efficient and low-cost method with faster analysis and with a smaller amount of sample. Based on the results obtained in this study, we believe that this technique has demonstrated great potential. In the future of virology, this technique may be developed for several viruses, and can be used as a screening technique, hence, being a powerful tool in viral diagnosis.

The results found in this study were encouraging. However, it is important to highlight that new studies must be carried out in order to improve the panel of samples used. That is, the investigation of a new panel of samples that includes positive aspects for several different viruses and, even, for other classes of microorganisms (as a negative control), must be undertaken.

## Biosecurity precautions

The procedures described in this study involving DENV and CHIKV were carried out in a level 2 biological safety laboratory according to decree 2,349 of the Brazilian Ministry of Health (https://bvsms.saude.gov.br/bvs/saudelegis/gm/2017/prt2349_22_09_2017.html) that adopted that CHIKV belongs to risk class 2; and the procedures for decontamination and sterilization of materials and equipment were made in accordance with the guidelines of the Laboratory Biosafety Manual of the World Health Organization^29^.

## Materials and methods

### Clinical samples

Sera from patients with suspected cases of infection by DENV and/or CHIKV were collected in several hospitals and health units in the state of Rio Grande do Norte/Brazil. Samples towards the uninfected class were collected from asymptomatic volunteers. Virus infected samples were collected during the viremic phase in a period of up to 1 year prior to spectral acquisition. Then, these samples were sent to the Laboratory of Molecular Biology of Infectious Diseases and Cancer or to the Laboratory of Virology of the Institute of Tropical Medicine of the Federal University of Rio Grande do Norte together with the patient notification form, and stored in a freezer – 70 °C until molecular analyses. Informed consent was obtained from all study participants. The Institutional Ethics Committee for Human Research of the Hospital Universitário Onofre Lopes (HUOL) based in the Federal University of Rio Grande do Norte (UFRN) (Brazil) approved this study (protocol number # 51057015.5.0000.5537) and informed consent was obtained from all subjects. Also, all the methods carried out in this study were by the approved guidelines.

### RNA extraction and reverse transcription-polymerase chain reaction

The RNA of the samples was extracted using the QIAmp Viral Mini Kit (Qiagen, Inc., Valencia, USA), according to the manufacturer’s instructions. To detect and type DENV, RT-PCR was performed following the protocol described by Lanciotti et al.^11^. For the diagnosis of CHIKV, the amplification of the genetic material occurred through qRT-PCR using the following primers: the probe VCHIK 6919P (100 nM) and the primers VCHIK 6856F (500 nM) and VCHIK 6981R (500 nM), according to the protocol described by Lanciotti et al.^12^. The absence of virus was also confirmed for samples constituting the uninfected class. The absence of co-infections with Zika or Yellow Fever was also confirmed by RT-qPCR.

### Sample preparation

To carry out this study, a total of 78 serum samples were used, in which 26 of them were from individuals confirmed with DENV, 26 from patients confirmed with CHIKV, and 26 from uninfected individuals, detected by the molecular techniques previously mentioned. For molecular fluorescence, a new methodology was proposed whereby mixtures of serum in milli-Q water are used. For this, 1,650 μL of milli-Q water were mixed with 50 μL of the serum sample in a 2 mL eppendorf, then they were vortexed for 10 s to obtain a homogeneous mixture. An optimization was made to accomodate the fluorescence signal intensity. That is, analyses were performed with different dilutions, and it was found that the best spectral signal was 1,650 µL of Milli-Q water + 50 µL of the sample. More dilute solutions tend to exhibit very weak signals < 100 units of intensity, whilst more concentrated mixtures tend to result in saturated signals (> 1,000 units of intensity). Finally, the resulting mixture was transferred to the 0.5 mm quartz cuvette, where the EEM fluorescence matrix was acquired.

### EEM fluorescence spectroscopy

For acquisition of the EEM matrices, the ambient temperature was maintained at 25 °C. A RF-5301 Shimadzu spetrofluorometer with a 0.5 mm quartz cuvette was used where 1,700 µL of the milli-Q water and serum mixture was added. The excitation/emission wavelength range was 250–320 nm for excitation and 240–800 nm for emission, with steps of 10 and 1 nm, respectively. Although the excitation range used is within the UV range, it is demonstrable that this does not cause problems in the classification process. The absorption of UV radiation causes photochemical damage to virus RNA since this radiation induces lesions such as cyclobutane pyrimidine dimers or 6–4 photoproducts, inhibiting virus replication^30^. However, since these lesions are minimal, occur only in viral RNA and in both virus types (DENV and CHIKV), this is not a problem from the standpoint of classification since the viral particles are still present in the sample, having just undergone inactivation. In addition, in this study there is a strong possibility that this inactivation has not occurred since the exposure time for inactivation is 90 s in UV. Herein, a 45-s exposure was used to obtain the EMM matrix. That is, 45 s to obtain the 8 emission spectra. This means that for each emission spectrum, the average excitation time was 5.62 s (5.62 s exposed to radiation of 250 nm, 5.62 s exposed to radiation of 260 nm and so on until 320 nm). Tseng and Li also proved that the relative humidity of the environment decreases the ability of UV radiation to inactivate the virus, since the water provides protection against UV-induced damage^31^. In our set-up, the viruses were in an aqueous medium, thus the ability of UV radiation to inactivate them is minimal. In summary, viral inactivation does not cause problems for the classification process, and the chances of this inactivation occurring are low.

The excitation and emission slits were maintained at 3 nm. The choice of excitation and emission slits was optimized in order to obtain a better signal-to-noise ratio and resolution*.* The scanning speed was set at 3,000 nm/min (super mode) in order to perform a faster spectral acquisition. After each acquisition, the cuvette was washed with milli-Q water, then with 70% (v/v) alcohol, and again with milli-Q water. Alcohol 70 is effective in disinfecting and consequently cleaning various materials, including the cuvette used in the experiment. In addition, it is also effective in eliminating viruses present in the same material. So as not to leave any traces of alcohol in the cuvette, the cleaning was completed with the addition of milli-Q water, to prevent ethanol becoming a component capable of degrading subsequent samples. Post-cleaning, the cuvette was allowed to dry completely to avoid dilution of subsequent samples.

### Software

The entire pre-processing and multiway classification procedure were done with MATLAB R2014b software (The Math-Works, Natick, USA), and the MATLAB toolbox for discriminant analysis based on trilinear three-way data, TTWD-DA 1.0^32^.

### Computational and chemometric procedure

As pre-processing, the Rayleigh and Raman scattering were removed using the 'EEMscat' algorithm^33^. Therefore, for the construction of the models, EEM matrices were used in a range of 250–320 nm for excitation with steps of 10 and 240–800 nm for emission with steps of 1, resulting in an array of data size 8 × 561 for each sample. The samples were divided into training (70%) and test (30%) sets using the Kennard-Stone sampling algorithm^34^. In the process of selecting samples for training and test sets, the samples are selected based on the calculation of the Euclidean distance, where the selected samples are those with maximum distances from all others, until the specified number of samples is reached for each set. Therefore, samples are selected to cover the entire sample space. Finally, the n-PLSDA, PARAFAC-LDA and PARAFAC-QDA classification methods were applied to the data cubes.

#### n-PLSDA

Several studies have addressed the application of partial least squares for classification purposes^35–39^. The n-PLSDA model is given in the following Eq. (1):

1 $${\mathrm{p}}_{\mathrm{ijk}}= \sum_{\mathrm{m}=1}^{\mathrm{M}}{\mathrm{t}}_{\mathrm{im}} {\mathbf{w}}_{\mathrm{jm}}^{\mathrm{J}} {\mathbf{w}}_{\mathrm{km}}^{\mathrm{K}}+ {\mathrm{e}}_{\mathrm{ijk}}$$

where ${p}_{ijk}$ is an instrumental response measured for a sample i in emission sensors j and excitation k (for the case of the EEM fluorescence matrix), ${\mathrm{t}}_{\mathrm{im}}$ is an element of the score matrix T, ${{\varvec{w}}}_{jm}^{J}$ and ${{\varvec{w}}}_{km}^{K}$ are elements related to the loadings associated with emission and excitation respectively, and ${e}_{ijk}$ is the model residue. The n-PLSDA model is an expansion of the known PLSDA, so the model finds the scores producing maximum covariance with the dependent variable, for three-dimensional data. Then, the scores for each sample are applied in Eq. (2), providing the codes of unknown samples.

2 $${\mathrm{c}}_{\mathrm{u}}= {\mathbf{t}}_{\mathrm{u}}^{\mathrm{T}}\mathbf{v}$$

with ${t}_{u}$ representing the scores of the samples in the test set, obtained by projecting the vectorized data in the latent factor space, given by:

3 $${\mathbf{t}}_{\mathbf{u}}= {\left({\mathbf{W}}^{\mathrm{T}}\mathbf{Q}\right)}^{-1}{\mathbf{W}}^{\mathrm{T}}({\mathbf{X}}_{\mathrm{u}})$$

The advantage of n-PLSDA over PLSDA is that Eq. (1) provides a stabilized decomposition, providing better predictions^40^.

#### PARAFAC

Parallel factor analysis (PARAFAC) is a well-known technique for bidirectional factor analysis for three-way data. PARAFAC is based on the idea that it is possible that the analysis of simultaneous factors of different matrices in parallel can lead to a single optimal set of factors^41^. A PARAFAC model of a three-way matrix (as for example for molecular fluorescence data where one path corresponds to the samples, another path to the emission spectra and the other path to the excitation spectra) is given by three loadings matrices (A, B and C) with their respective elements ${a}_{if}$, ${b}_{jf}$ and ${c}_{kf}$. The model uses the criterion of measuring the sum of squares of the residuals ${e}_{ijk}$ according to Eq. (4), as follows:

4 $${\mathbf{x}}_{\mathrm{ijk}}= \sum_{\mathrm{f}=1}^{\mathrm{F}}{\mathbf{a}}_{\mathrm{if}} {\mathbf{b}}_{\mathrm{jf}} {\mathbf{c}}_{\mathrm{kf}}+ {\mathbf{e}}_{\mathrm{ijk}}$$

The PARAFAC model can also be represented as in Eq. (5)

5 $$\underset{\_}{X}= \sum_{\mathrm{f}=1}^{\mathrm{F}}{\mathbf{a}}_{\mathrm{f}} \otimes {\mathbf{b}}_{\mathrm{f }}\otimes {\mathbf{c}}_{\mathrm{f}}$$

where ${\mathbf{a}}_{\mathrm{f}}$, ${\mathbf{b}}_{\mathrm{f}}$ and ${\mathbf{c}}_{\mathrm{f}}$ are the columns of the loading matrices A, B and C, respectively (${\mathbf{a}}_{\mathrm{f}}$ can also be called scores)^42–44^. In classification analysis, PARAFAC acts as an exploratory analysis for intelligent data reduction, providing scores ${\mathbf{a}}_{\mathrm{f}}$, or loadings ${\mathbf{b}}_{\mathrm{f}}$ and ${\mathbf{c}}_{\mathrm{f}}$, which can be used for classification purposes by applying linear discriminant analysis (LDA) or quadratic discriminant analysis (QDA). An important application of PARAFAC is that it seeks to retrieve the individual profiles of the species that provide fluorescence signals, and these profiles can be observed in the excitation and emission loadings.

#### LDA

LDA is a supervised classification technique capable of maximizing differences between samples of different classes, and minimizing differences between samples of the same class. For this, the LDA calculates the classification score considering that all classes have the same covariance matrix^45^. This calculation is done according to Eq. (6):

6 $${\mathrm{L}}_{\mathrm{ik}}= {({\mathbf{x}}_{\mathrm{i}}- {\stackrel{-}{\mathbf{x}}}_{\mathrm{k}})}^{\mathrm{T}} {\sum }_{\mathrm{pooled}}^{-1}({\mathbf{x}}_{\mathrm{i}}- {\stackrel{-}{\mathbf{x}}}_{\mathrm{k}}){({\mathbf{x}}_{\mathrm{i}}- {\stackrel{-}{\mathbf{x}}}_{\mathrm{k}})}^{\mathrm{T}}-2{log}_{e}{\pi }_{k}$$

where ${\mathbf{x}}_{\mathrm{i}}$ represents an unknown vector for a sample i; ${\stackrel{-}{\mathbf{x}}}_{\mathrm{k}}$ represents an average vector of class k; ${\sum }_{pooled}$ is the pooled covariance matrix; and ${\pi }_{k}$ is the prior probability of class k^24^.

#### QDA

QDA is a distance measurement similar to the measurement made by LDA. However, unlike LDA, QDA considers a sample variance–covariance matrix for each class, according to Eq. (7)^45^:

7 $${Q}_{ik}= {({\mathbf{x}}_{\mathrm{i}}- {\stackrel{-}{\mathbf{x}}}_{\mathrm{k}})}^{\mathrm{T}} {\sum }_{\mathrm{k}}^{-1}({\mathbf{x}}_{\mathrm{i}}- {\stackrel{-}{\mathbf{x}}}_{\mathrm{k}})+ {log}_{e} \left|{\sum }_{k}\right|-2{log}_{e} {\pi }_{k}$$

where ${\sum }_{k}$ is the variance–covariance matrix of class k; ${log}_{e} \left|{\sum }_{k}\right|$ is the natural logarithm of the variance–covariance matrix ${\sum }_{k}$^24^.

### Quality performance

As a measure of the quality of the classification results, correct classification rate (CC%, Eq. 8), accuracy (AC, Eq. 9), sensitivity (S, Eq. 10), specificity (SP, Eq. 11) and F score (Eq. 12) were used as merit figures.

8 $$CC\%=100- \frac{\left({\varepsilon }_{1}- {\varepsilon }_{2}\right)}{N}\times 100$$

9 $$AC= \frac{(TP+TN)}{TP+TN+FP+FN}$$

10 $$S= \frac{TP}{TP+FN } \times 100$$

11 $$SP= \frac{TN}{TN+FP} \times 100$$

12 $$F-score= \frac{2 \times S \times SP}{S+SP}$$

The CC% represents the percentage of samples correctly classified considering their true classes. For this, it uses a binary approach, where ${\varepsilon }_{1}$ represents the errors for class 1 (class of interest) and ${\varepsilon }_{2}$ represents the errors for the class 2 (all samples that do not belong to class 1). The CC% is calculated for all stages (training and testing) and N represents the total number of samples used in that step; the AC informs the number of correct answers, considering true and false negatives; S informs the percentage of positive samples matched by the model; SP informs the percentage of negative samples that were hit by the model; and the F score represents the overall performance of the classification considering unbalanced data. TP, TN, FP and FN mean true positive, true negative, false positive and false negative, respectively^25^.

## References

[1] Boga JA (2019). Simultaneous detection of dengue virus, chikungunya virus, zika virus, yellow fever virus and west nile vírus. J. Virol. Methods.

[2] Guzman MG (2010). Dengue: A continuing global threat. Nat. Rev. Microbiol..

[3] Febre Chikungunya: Manejo clínico, Ministério da saúde https://www.saude.gov.br/images/pdf/2017/maio/31/chikungunya_manejo_clinico_2017.pdf (2017).

[4] Furuya-Kanamori L (2016). Co-distribution and co-infection of chikungunya and dengue viruses. BMC Infect. Dis..

[5] Boonham N (2014). Methods in virus diagnostics: From ELISA to next generation sequencing. Virus Res..

[6] Dejnirattisai W (2016). Dengue virus sero-cross-reactivity drives antibody-dependent enhancement of infection with zika virus. Nat. Immunol..

[7] Kawiecki AB, Christofferson RC (2016). Zika-induced antibody response enhances dengue serotype 2 replication in vitro. J. Infect. Dis..

[8] Cabral-Castro MJ, Cavalcanti MG, Peralta RHS, Peralta JM (2016). Molecular and serological techniques to detect co-circulation of DENV, ZIKV and CHIKV in suspected dengue-like syndrome patients. J. Clin. Virol..

[9] Araújo JMG, Bello G, Schatzmayr HG, Santos FB, Nogueira RMR (2009). Dengue virus type 3 in Brazil: A phylogenetic perspective. Mem. Inst. Oswaldo Cruz..

[10] Guzman MG, Kourí G (2004). Dengue diagnosis, advances and challenges. Int. J. Infect. Dis..

[11] Lanciotti RS, Calisher CH, Gubler DJ, Chang G, Vorndam AV (1992). Rapid Detection and typing of dengue viruses from clinical samples by using reverse transcriptase–polymerase chain reaction. J. Clin. Microbiol..

[12] Lanciotti RS (2007). Chikungunya virus in US travelers returning from India, 2006. Emerg. Infect. Dis..

[13] Plourde AR, Bloch EM (2016). A literature review of zika virus. Emerg. Infect. Dis..

[14] Hayes EB (2009). Zika virus outside Africa. Emerg. Infect. Dis..

[15] Lindsey HS, Calisher CH, Mathews JH (1976). Serum dilution neutralization test for California group virus identification and serology. J. Clin. Microbiol..

[16] Sousa, A. R. V. Avaliação sorológica e molecular de pacientes com quadro clínico de dengue símile atendidos no hospital das forças armadas. https://repositorio.bc.ufg.br/tede/bitstream/tede/9468/5/Disserta%C3%A7%C3%A3o%20-%20Adriano%20Roberto%20Vieira%20de%20Sousa%20-%202019.pdf (2019).

[17] Tool for the diagnosis and care of patients with suspected arboviral diseases, Pan American Health Organization. https://iris.paho.org/xmlui/bitstream/handle/123456789/33895/9789275119365_eng.pdf?sequence=1&isAllowed=y (2017).

[18] Santos MCD (2018). ATR-FTIR spectroscopy with chemometric algorithms of multivariate classification in the discrimination between healthy vs dengue vs chikungunya vs zika clinical samples. Anal. Methods..

[19] Santos MCD, Nascimento YM, Araújo JMG, Lima KMG (2017). ATR-FTIR spectroscopy coupled with multivariate analysis techniques for the identification of DENV-3 in different concentrations in blood and serum: A new approach. RSC Adv..

[20] Stedmon CA, Bro R (2008). Characterizing dissolved organic matter fluorescence with parallel factor analysis: A tutorial. Limnol. Oceanogr. Methods..

[21] Costa FSL (2017). Comparison of multivariate classification algorithms using EEM fluorescence data to distinguish Cryptococcus neoformans and Cryptococcus gattii pathogenic fungi. Anal. Methods..

[22] Conklin MW, Provenzano PP, Eliceiri KK, Sullivan R, Keely PJ (2009). Fluorescence lifetime imaging of endogenous fluorophores in histopathology sections reveals differences between normal and tumor epithelium in carcinoma in situ of the breast. Cell Biochem. Biophys..

[23] Teixeira AP (2009). In situ 2D fluorometry and chemometric monitoring of mammalian cell cultures. Biotechnol. Bioeng..

[24] Siqueira LFS, Júnior RFA, Araújo AA, Morais CLM, Lima KMG (2017). LDA vs QDA for FT-MIR prostate cancer tissue classification. Chemom. Intell. Lab. Syst..

[25] Santos MCD, Morais CLM, Nascimento YM, Araujo JMG, Lima KMG (2017). Spectroscopy with computational analysis in virological studies: A decade (2006–2016). TrAC..

[26] Skoog DA, West DM, Holler FJ, Crouch SR (2017). Fundamentals of Analytical Chemistry.

[27] Peck K, Stryer L, Glazer AN, Mathies RA (1989). Single-molecule fluorescence detection: Autocorrelation criterion and experimental realization with phycoerythrin. Proc. Natl. Acad. Sci. USA.

[28] Peeling RW (2010). Evaluation of diagnostic tests: Dengue. Nat. Rev. Microbiol..

[29] Laboratory Biosafety Manual: 3rd Edition, World Health Organization. https://www.who.int/csr/resources/publications/biosafety/Biosafety7.pdf?ua=1 (2004). 24404640

[30] Mathew AM, Mun AB, Balakrishnan A (2018). Ultraviolet inactivation of chikungunya virus. Intervirology.

[31] Tseng C, Li C (2005). Inactivation of virus-containing aerosols by ultraviolet germicidal irradiation. Aerosol. Sci. Technol..

[32] Morais CLM, Lima KMG, Martin FL (2019). TTWD-DA: A MATLAB toolbox for discriminant analysis based on trilinear three-way data. Chemom. Intell. Lab. Syst..

[33] Bahram M, Bro R, Stedmon C, Afkhami A (2006). Handling of Rayleigh and Raman scatter for PARAFAC modeling of fluorescence data using interpolation. J. Chemom..

[34] Kennard RW, Stone LA (1969). Computer aided design of experiments. Technometrics.

[35] Morais CLM, Santos MCD, Lima KMG, Martin FL (2019). Improving data splitting for classification applications in spectrochemical analyses employing a random-mutation Kennard–Stone algorithm approach. Bioinformatics.

[36] Barker M, Rayens W (2003). Partial least squares for discrimination. J. Chemom..

[37] Breretona RG, Lloyd GR (2014). Partial least squares discriminant analysis: Taking the magic away. J. Chemom..

[38] Gromski PS (2015). A tutorial review: Metabolomics and partial least squares-discriminant analysis—a marriage of convenience or a shotgun wedding. Anal. Chim. Acta.

[39] Ouertani SS, Mazerolles G, Boccard J, Rudaz S, Hanafi M (2014). Multi-way PLS for discrimination: Compact form equivalent to the tri-linear PLS2 procedure and its monotony convergence. Chemom. Intell. Lab. Syst..

[40] Azcarate SM, Gomes AA, Peña AM, Goicoechea HC (2018). Modeling second-order data for classification issues: Data characteristics, algorithms, processing procedures and applications. TrAC..

[41] Harshman RA, Lundy ME (1994). PARAFAC: Parallel factor analysis. Comput. Stat. Data Anal..

[42] Bro R (1997). PARAFAC. Tutorial and applications. Chemom. Intell. Lab. Syst..

[43] Andersen CM, Bro R (2003). Practical aspects of PARAFAC modeling of fluorescence excitation–emission data. J. Chemom..

[44] Murphy KR, Stedmon CA, Graeberc D, Bro R (2013). Fluorescence spectroscopy and multi-way techniques PARAFAC. Anal. Methods..

[45] Dixon SJ, Brereton RG (2009). Comparison of performance of five common classifiers represented as boundary methods: Euclidean distance to centroids, linear discriminant analysis, quadratic discriminant analysis, learning vector quantization and support vector machines, as dependent on data structure. Chemom. Intell. Lab. Syst..

